# What’s keeping people after stroke from walking outdoors to become physically active? A qualitative study, using an integrated biomedical and behavioral theory of functioning and disability

**DOI:** 10.1186/s12883-016-0656-6

**Published:** 2016-08-15

**Authors:** Jacqueline Outermans, Jan Pool, Ingrid van de Port, Japie Bakers, Harriet Wittink

**Affiliations:** 1 Research Group Lifestyle and Health, Research Centre for Innovations in Healthcare, Hogeschool Utrecht University of Applied Sciences, Heidelberglaan 7, 3584CS Utrecht, The Netherlands; 2 Revant, Brabantlaan 1, 4817JW Breda, The Netherlands; 3 Utrecht University Medical Centre, Heidelberglaan 100, 3584CX Utrecht, The Netherlands

**Keywords:** Stroke, Community ambulation, Outdoor walking, Physical activity

## Abstract

**Background:**

In general people after stroke do not meet the recommendations for physical activity to conduct a healthy lifestyle. Programs to stimulate walking activity to increase physical activity are based on the available insights into barriers and facilitators to physical activity after stroke. However, these programs are not entirely successful. The purpose of this study was to comprehensively explore perceived barriers and facilitators to outdoor walking using a model of integrated biomedical and behavioral theory, the Physical Activity for people with a Disability model (PAD).

**Methods:**

Included were community dwelling respondents after stroke, classified ≥ 3 at the Functional Ambulation Categories (FAC), purposively sampled regarding the use of healthcare. The data was collected triangulating in a multi-methods approach, i.e. semi-structured, structured and focus-group interviews. A primarily deductive thematic content analysis using the PAD-model in a framework-analysis’ approach was conducted after verbatim transcription.

**Results:**

36 respondents (FAC 3–5) participated in 16 semi-structured interviews, eight structured interviews and two focus-group interviews. The data from the interviews covered all domains of the PAD model. Intention, ability and opportunity determined outdoor walking activity. Personal factors determined the intention to walk outdoors, e.g. negative social influence, resulting from restrictive caregivers in the social environment, low self-efficacy influenced by physical environment, and also negative attitude towards physical activity. Walking ability was influenced by loss of balance and reduced walking distance and by impairments of motor control, cognition and aerobic capacity as well as fatigue. Opportunities arising from household responsibilities and lively social constructs facilitated outdoor walking.

**Conclusion:**

To stimulate outdoor walking activity, it seems important to influence the intention by addressing social influence, self-efficacy and attitude towards physical activity in the development of efficient interventions. At the same time, improvement of walking ability and creation of opportunity should be considered.

## Background

In the Netherlands approximately 220 thousand stroke survivors, as part of a population of 17 million inhabitants, suffer from more or less severe functional impairments [1]. Although 39–85 % of the stroke survivors attain an independent level of walking [2, 3], it has been shown that 26 % of home dwelling stroke patients show no or limited walking activity three years after inpatient rehabilitation due to stroke [4, 5]. A meta-analysis [6] showed that among 1105 people, between 3 months to 8,5 years after stroke, a mean of 4355 steps a day were taken, which is well below the current recommendation for people with a disability of 6500–8500 steps a day [7]. This inactive lifestyle may perpetuate existing impairments and deconditioning. Deconditioning, resulting in low levels of physical fitness, specifically aerobic capacity, has been recognized as a major problem in stroke [8]. It is associated with health risks such as metabolic syndrome, cardiovascular disease or recurrent stroke [8, 9] as well as with reduced walking capacity [10]. Evidence for benefits of increased physical activity on health in stroke is getting stronger [11] although it is not yet clear if it also reduces recurrent stroke risk. Furthermore, moderate to vigorous walking interventions on a treadmill were shown effective in improving aerobic capacity after stroke [12].

Therefore, it seems paramount to establish effective programs to stimulate outdoor walking to become physically active. Being physically active has been defined as “meeting established guidelines for physical activity, that are activities of at least moderate intensity” [13]. To accomplish that, knowledge about perceived barriers and facilitators specifically to outdoor walking aimed at staying or becoming physically active and reduce health risks is needed. However, many of the patient perceptions of barriers and facilitators that have been reported seem to be focused on community ambulation [14], travelling outdoors [15, 16] or physical activity in general [17]. Barriers and facilitators such as self-efficacy, beliefs about physical activity, self-determination and social support as well as ongoing professional support have been identified [14–17]. However, as the purpose of community ambulation and travelling outdoors may lay within the domain of participation International Classification of Functioning, Disability and Health (ICF) [18], the purpose of being physically active lies primarily within the ICF domain of activities with the specific goal of reduction of health risks or conducting a healthy lifestyle. Therefore, barriers and facilitators to being physically active may differ from those to community ambulation or traveling outdoors. Other studies [19, 20] explored patient perceptions influencing participation in structured exercise programs, being a subset of physical activity [21] after stroke. These studies showed that people after stroke have a preference for group exercise in a structured and dependent manner [19] and found that perceived impairments, lack of motivation and availability of facilities to exercise were barriers to exercise [20]. Exercise facilitators were social support from professionals and peers and planned activities to fill daily schedules. However, similar to community ambulation and outdoor traveling, the purpose of exercise, i.e. improvement of physical fitness [21], primarily lying within the ICF domain of body function and structures, is different from the purpose of becoming physically active. Again, barriers and facilitators may therefore differ.

Moreover, programs designed in the last decade to improve physical activity and community ambulation after stroke have not been successful [22, 23]. Interventions such as supervised exercise [24], lifestyle counseling [25], repeated instructions [26] or supervised outdoor walking [27–29] did not increase the level of physical activity after stroke. One explanation could be that many studies on barriers and facilitators, that form the foundation of programs to improve physical activity to date, either only used or developed behavioral theory [22] or only used the ICF. No comprehensive approach integrating these models has been undertaken to date. Johnston and Dixon [30] suggest that models integrating the ICF with behavioral models are more effective in explaining functional behavior than the ICF or behavioral theory separately.

Van der Ploeg and colleagues [31] proposed the Physical Activity for people with a Disability model (PAD-model), which integrates the Attitude, Social influence and self-Efficacy (ASE) model [32], which is based on the Theory of Planned Behavior (TPB) [33, 34], with the ICF model. We hypothesized that the PAD-model would provide a comprehensive overview of behavioral and physical barriers and facilitators for outdoor walking to increase physical activity. To our knowledge there is no study that explored the usefulness of this model in a stroke population.

The first aim of this study was to establish the barriers and facilitators from the perspective of Dutch home dwelling individuals after stroke in the chronic stage to outdoor walking to be physically active. The second aim was to determine the usefulness of the PAD model to generate a comprehensive overview of barriers and facilitators.

## Methods

### Design

This study employed qualitative methodology to ensure that the experiences and views of the participants would be identified so that perceived barriers and facilitators to walking outdoors and their meaning among a group of community dwelling stroke survivors could be better understood.

The first researcher (JO) was a physical therapist with 25 years of experience in neurological rehabilitation. The second researcher (SL), who participated in the analysis, was a 4th year student of the bachelor program in physical therapy, who had minor experience in neurological rehabilitation. The third researcher (JB) was a physical therapist with 5 years of experience in neurological rehabilitation. Two more researchers (JP and HW) with ample experience in conducting research completed the research team. All researchers were familiar with the PAD-model and as clinicians experienced in using the ICF in clinical reasoning.

### Respondent recruitment

To recruit respondents for the individual interviews, an existing network of physical therapy practices and daycare departments of nursing homes was used. To increase representativeness, purposive sampling was used with respect to healthcare utilization as this was expected to influence walking activity. Respondents should either 1) utilize daycare facilities two or more days per week, or 2) visit their physical therapy private practice once or twice a week or 3) not use physical therapy regularly.

Inclusion criteria were; community dwelling people in the wider urban region of the city of Utrecht, the Netherlands, with 1) a diagnosed stroke, as defined by the World Health Organization (WHO) [35] and 2) ability to walk independently with supervision if needed, categorized as functional ambulation categories (FAC) ≥3 [36]. Exclusion criterion was the inability to understand spoken or written language as a result from receptive aphasia defined as a score of ≤ 3 points using the Utrecht Communication Assessment (UCA) [37].

Potential respondents for the individual interviews were made aware of the study by their attending physical therapists or district nurse and registered if they were interested to participate. An information letter and informed consent form were subsequently sent to be signed by the potential respondent. Thereafter the researcher scheduled an appointment with the respondent at their homes.

Two focus-group interview sessions were organized during the monthly support meeting of the local group of the Dutch stroke patients’ organization using convenience sampling. Inclusion criteria were the same as used for the individual interviews. Prior to the focus-group interview the entire group was informed and thereafter the group members who wanted to participate signed informed consent forms.

### Data collection

A topic list to guide through the interviews was developed using the PAD-model [31] as a sensitizing concept (Table 1).

**Table 1:** Topic list semi-structured and structured individual interviews (phase 1) and focus-group interview (phase 2)

*Topics for all interviews*		*Choice of answers only for structured interview*				
Topic 1: Walking for health	What’s your opinion on your health situation?	Bad health	Not so healthy	Fair	Good	Don’t know
	What’s your opinion on your walking activity?	Little	Fair	Good	Very good	Don’t care
	Is walking of influence on your health?	No	Not really	A little positively	Positively	Negatively
	Is your health situation of influence on your walking activities?	No	Not really	A little	Yes	Don’t know
Topic 2: Exercise and physical activity	Did you participate in any sports or physical activity prior to your stroke?	No	Not really	A little	Yes	
	Is physical activity important to you?	Not at all	A little	Important	Very important	
	Are you currently participating in physical activity programs or exercise programs?	No	At the physical therapist’	At the sports club, the gym	By myself	
	When not, would you like to?	Yes, very much	Yes	Not really	No	
	What’s keeping you?	Afraid, dangerous	Physically not possible	Not in the mood	Has no purpose	Have done enough
	What’s driving you?	Keeping mobile and healthy	Just want to exercise	Partner/healthcare professional says to	Meeting other people	Want to get out
Topic 3: Walking outdoors	Do you walk outdoors each day?	Every day	2-3 times a week	Once a week	Almost never	More than once a day.
	What are your reasons for walking outdoors?	Exercise	Just for fun, getting some fresh air	Meeting with friends	Running errands	
	What’s keeping you from walking outdoors?	Uneven surfaces, crowds and obstacles	When there is no purpose to go outdoors	Have other means of transportation	Problems with orientation, motor control, balance or endurance.	Not allowed to go by myself, not safe
	How do you cope with problems when walking outdoors?	Avoid them	Encounter them	Ask assistance	Don’t know	
	What stimulates you to walk outdoors?	Walking with peers	Nice weather	Necessity to go	Stimulating caregiver	Stimulating healthcare professional

After the first four individual semi-structured interviews, a structured interview form was created to use with the respondents that suffered from expressive aphasia (UCA > 3). The topic list was identical to the one that was used in the semi-structured interviews. Each question had a choice of answers generated from the results of the first four semi-structured interviews as shown in Table 1. This enabled the respondents suffering from expressive aphasia to participate and they were encouraged to elaborate on their answer of choice to the best of their abilities.

The data was collected triangulating in a multi-methods approach, i.e. semi-structured, structured and focus-group interviews to increase the validity and rigor of the methods of the study. During the first phase of the study, the individual semi-structured interviews as well as the structured interviews were continued until there appeared to be saturation of data. Thereafter, in the second phase, two focus group sessions were performed. The focus-group interviews were used to confirm the saturation of the earlier collected data and as a means to validate these data. The respondents who participated in the individual interviews were different from the respondents whom participated in the focus group sessions. To increase the reliability of the collected data all semi-structured interviews and focus-group interviews were audio recorded. The structured interviews were not audio recorded to create a safe enough environment for respondents that suffered from expressive aphasia, allowing them to speak freely according to their ability. During all interviews field notes were taken.

To increase ecological validity, the individual interviews were conducted at the respondents’ homes. Family members, when present, were allowed to stay in the interviewing room. They were requested not to participate in the interview, unless they felt that important information would be missed. The same researcher who performed the interviews (JO) moderated the focus-group interview sessions. Each individual interview as well as the focus-group interviews lasted approximately 40 min.

### Data analysis and synthesis

Recordings were transcribed verbatim by research assistants and to verify their accuracy, one researcher (JO) independently checked the transcriptions.

A primarily deductive thematic content analysis, driven by the PAD model as directing concept, was performed using the five-stage ‘Framework’ approach [38, 39]. Stages of analysis included: (1) familiarization, (2) thematic framework development, (3) indexing, (4) charting, and (5) mapping and interpretation. The analysis was performed in Excel (Microsoft Office 2013).

The first stage involved repeated listening to and reading of the transcripts and collected field notes in order to become familiar with the data. During this stage, notes were taken on the recurrent themes and issues that emerged from the PAD model, keeping an open mind, however, to other emerging themes. In the second stage, the PAD model served as a theoretical framework to provide a priori determined key issues and concepts. Accordingly, a thematic framework was developed in which we explicated normative beliefs, control beliefs and behavioral beliefs originating from the TPB [33, 34], underlying social influence, self-efficacy and attitude respectively in the ASE-model [32], to be able to sort the data.

The third stage was used to systematically apply the developed thematic framework to the data. All information from the transcripts that was relevant to each index heading was copied into the framework to build a descriptive overview for all headings. The fourth stage involved producing a summary of the respondents' views or experiences under each heading. During the final stage, the charts were reviewed systematically in order to detect patterns or associations within the data.

Two researchers (JO and SL) analyzed the individual interviews and two researchers (JO and JB) analyzed the data from the focus-group interviews. To increase the reliability and rigor of the analysis a consensus meeting was scheduled after each stage of the analysis. Furthermore, peer-debriefing sessions were conducted between three researchers (JO, JP and HW) in the fifth stage of analysis.

## Results

A total of 36 home dwelling respondents, participated in the study. Table 2 shows that 15 respondents participated in the individual semi-structured interviews, eight respondents in the individual structured interviews and a total of 13 respondents in the two focus-group interviews. Mean age of the respondents was 70.8 years ranging from 46 to 89 years. Twenty-one (57 %) respondents were male, of which 14 (67 %) were married. Fifteen respondents were female, of which 6 (40 %) were married. Seventeen respondents received daycare at a facility at least 2 times a week, 11 respondents received physical therapy treatment once or twice a week and eight respondents did not receive physical therapy regularly. Eight respondents were able to walk independently, but needed supervision. They were categorized into FAC 3. Eight respondents reached FAC 4, being able to negotiate all surfaces when even. Twenty (56 %) respondents were able to walk on any, including uneven, surfaces, FAC 5. Seventeen (53 %) respondents used assistive devices for walking. Ten respondents used a rollator and 7 used a cane.

**Table 2:** Characteristics of the respondents

	Phase 1		Phase 2	
	Semi-structured interview *n* = 15	Structured interview *n* = 8	Focus group A *n* = 7	Focus group B *n* = 6
Age (y)				
Mean (SD)	71.3 (13.3)	72.5 (8.8)	69.3 (9.2)	69.2 (10.3)
Range	(46-89)	(60-83)	(52-81)	(57-82)
Gender				
Male (%)	8 (53 %)	7 (88 %)	2 (29 %)	4 (67 %)
Marital status				
Married (%)	9 (60 %)	3 (38 %)	4 (57 %)	4 (60 %)
Utilisation of healthcare (%)	4 PT (27 %) 2 No regular PT (13 %) 9 Daycare (60 %)	3 PT (38 %) 5 Daycare (62 %)	2 PT (29 %) 4 No regular PT (57 %) 1 Daycare (14 %)	2 PT (33 %) 2 No regular PT (33 %) 2 Daycare (33 %)
FAC (%)	5 FAC3 (33 %) 1 FAC4 (7 %) 9 FAC 5 (60 %)	3 FAC3 (38 %) 3 FAC4 (38 %) 2 FAC5 (24 %)	2 FAC4 (29 %) 5 FAC5 (71 %)	2 FAC4 (33 %) 4 FAC5 (67 %)
Assistive devices (%)	1 cane (7 %) 7 rollator (46 %)	2 cane (25 %) 3 rollator (38 %)	2 cane (29 %)	2 cane (33 %)

Abbreviations: *y* years, *SD* standard deviation, *FAC* Functional Ambulation Categories, *PT* physical therapy

**Fig. 1:**
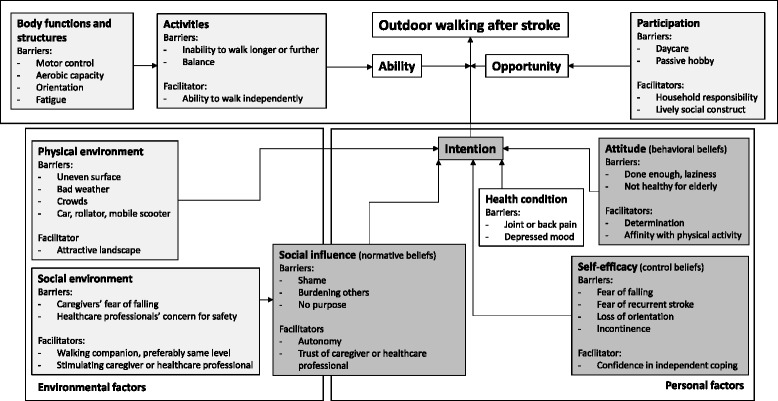
The PAD model adapted to outdoor walking after stroke. The paler grey rectangles depict the ICF; the darker grey rectangles show the ASE model

The data covered all domains of the PAD model [31] that was used. This is shown in Fig. 1. Using the PAD-model three main categories were identified: 1). the intention to walk outdoors, 2). the ability to walk outdoors and 3). the opportunity to walk outdoors. The intention to walk outdoors results from the attitude and self-efficacy towards outdoor walking as well as social influence. Social and physical environment furthermore influence the intention to walk outdoors, where social environment seems to have a direct link to social influence and physical environment to self-efficacy as shown in Fig. 1. The ability to walk outdoors consists of the ability to walk far enough and to maintain a standing posture. These abilities are influenced by body functions. The opportunity to walk outdoors is linked to occupational and leisure activities at the level of participation in the ICF.

### Facilitators and barriers for the intention to walk outdoors identified from the PAD model

Behavioral beliefs underlying the attitude towards walking, such as having walked enough over the life span as well as brisk walking being unhealthy for elderly were identified as barriers. As a 75-year-old respondent commented: *“I constantly come home more tired than when I left, that can’t be right, can it? From exercise? I do not think so; it was too much. I felt my heart beat too quickly, that can’t be good for me at my age? I did not like it very much.”*

Behavioral beliefs such as determination to walk and having affinity with physical activity as a healthy lifestyle were perceived as facilitators for walking outdoors. Illustrated by a respondents’ view: *“I do not always particularly feel like it, but I think I should walk at least a little every day, I just have that feeling I should stay limber…because I know exercise is good for me”*

Normative beliefs underlying social influence such as “walking outdoors has to be for a purpose”, for instance, to go the grocery store could be a barrier to walk outdoors to increase physical activity. Expressed by a female respondent as*: “There is nothing I dislike more than walking for no purpose.”*

Being ashamed of the decreased ability to walk or being accompanied by a much better walker was perceived a barrier to outdoor walking, formulated by a respondent as*: “No, in the beginning they walked with me, but I prefer to go alone. I feel like I am in the way. I am fine walking by myself.”*

Barriers to outdoor walking that were identified in the social environment were the caregivers’ fear of falls and the healthcare professionals’ primary concern for safety. Facilitators at this level were having a walking companion or a stimulating caregiver or healthcare professional. Facilitators had a positive impact on social influence in turn leading to a positive intention to walk outdoors. As a respondent said*: “But first I must have my confidence back and my wife also, because she saw me fall twice and had to help me. So you do not want to wait for it to happen a third time.”* Or another respondents’ comment*: “Yes, yes, because at the daycare center I walk without a cane and I did well. Last Tuesday there was a new physical therapist asking where I had left my cane. He was pretty anxious, more so than me, because I’m walking without the cane all the time.”*

Control beliefs underlying self-efficacy such as low falls efficacy, were identified as a barrier to outdoor walking. One respondent said*: “No I am not afraid or anything, but walking is just more complicated. Perhaps you think all it takes is a little push from someone or other and I am down. I’d like to avoid that, of course.”* Similarly, the view of another respondent*: “If you tell me to go to the market with my rollator I’d tell you to go yourself. You know, they all are constantly running you off your feet.”* Furthermore, fear of recurrent stroke and loss of orientation as well as incontinence were identified as barriers. The belief to be able to cope independently or that an accompanying person would be able to cope in case of adverse events such as a fall seemed to facilitate outdoor walking. As a 50-year old respondent said: *“No, I am limber enough not to fall like a log.”*

Joint pain, such as back pain, was indicated as a barrier to outdoor walking, illustrated by this comment*: “Well yes, when I walk my back starts hurting me and then I think I am not going to walk anymore, I can’t walk anymore.”* This barrier, accompanied with depressed mood, had a negative influence on both self-efficacy and attitude towards outdoor walking and thereby on the intention to walk outdoors.

Barriers in the physical environment were uneven surfaces outdoors and bad weather.

A single living respondent commented on that: *“Yes, obviously the weather is very important. I am not fond of walking in storms and rain, but nothing much else prevents me from walking. If I want or need to walk, I go!”* Furthermore, crowds and conveniences such as the availability of a car, mobility scooter were barriers as well as the presence of a freezer, which reduced the necessity to go out for groceries. One respondent, who used a rollator said: *“…let’s be honest, I have a mobility scooter that I love. Why would I walk with my rollator? You can only use that for exercise around the house perhaps, but nothing much else.”* An attractive landscape and the availability of assistive devices such as canes or rollators were identified as a facilitator for outdoor walking. Illustrated by the following remark: *“And because of that I kept falling to the right, but without harm. I could get up myself with the help of my rollator.”*

Barriers in the physical environment negatively influenced self-efficacy in turn reducing the intention to walk outdoors.

### Facilitators and barriers for the ability to walk outdoors identified from the PAD model

The barriers for walking outdoors at the level of body functions and structures were impaired cognitive function, e.g. memory, as well as reduced motor control and postural or balance reactions as a result of the hemiplegia, strength and aerobic capacity. One respondent, who used daycare: “*I say I have a leg that doesn’t work. It causes one to shuffle. Can’t lift it anymore.”* Aerobic capacity was indicated as a barrier as another respondent in daycare said: *“I’ll sit down on my rollator for a little while, because it is quite a distance and walking far is very difficult for me, I totally get out of breath.”* Furthermore, fatigue was mentioned by one respondent*: “Isn’t it strange, when I do nothing I am still tired.”*

A barrier that was identified on the level of activities was the inability to walk longer distances, illustrated by an independently walking respondent: *“I’m partially paralyzed, so it is always difficult. But even with a cane I can walk only for 5 to 7 min.”* Also inability to uphold balance was identified. Facilitators at this level were the ability to walk independently.

### Facilitators and barriers for the opportunity to walk outdoors identified from the PAD model

Facilitators at the level of participation enhanced the positive intention for outdoor walking such as responsibilities in household tasks demanding walking, like shopping for groceries as a married respondent mentioned*: “When we are out of bread, I’m the one who walks to the market to get new supplies”* On the other hand, daycare offers little opportunity for walking outdoors like a respondent said:*” On the days that I am in the daycare facility, there’s nothing much to do except for one half hour of physical therapy. We sit most of the time playing games and talking, drinking coffee or in the afternoon a small snifter”.*

## Discussion

The first aim of the study was to give insight into perceived barriers and facilitators in all domains of the PAD-model describing outdoor walking activity to become physically active in individuals after stroke. Overall, outdoor walking activity seems to be a result of the intention to walk, walking ability and opportunity to walk. The intention to walk outdoors was determined by the perceived barriers and facilitators in social influence, self-efficacy and attitude with underlying environmental factors, i.e. social and physical environment. Social influence seemed impacted by social environment, which consequently influenced the intention to walk. For example, the respondents stated that they often felt inhibited by their caregivers, who felt it to be unsafe for them to walk outdoors. Additionally, they felt held back by their professional caregivers, as they seemed more concerned with safety than with improvement of physical activity, which was also reported in a hospital setting [40]. The cautiousness of caregivers and professionals has also been reported in studies on stimulating traveling outdoors early after stroke [15] and on physical activity in general in chronic stroke [41]. The intention to walk outdoors was positively influenced by opportunities that derived from participation such as hobbies, social activities and household responsibilities. For example, the respondents in the present study who were living alone or whose spouses did not take the household responsibility, all reported that the need to go out for groceries enhanced their walking activity. Conversely, the ones living with a partner that took all responsibilities felt no urgency to get out and about. These determinants are much like the reasons reported for resuming valued activities after stroke [42]. The barriers and facilitators, such as purposefulness and perceived burden on companions or caregivers that constructed social influence and lead up to intention, were in line with several other studies on physical activity [41], other valued activities [42] and travelling outdoors even early after stroke [15].

The ability to walk a reasonable distance and the ability to maintain balance were perceived as determinants for outdoor walking ability with underlying impairments of body functioning such as strength and aerobic capacity. Balance has previously been identified as an important barrier in line with studies that focused on barriers and facilitators for exercise [43] and resuming valued activities [42]. Physical and cognitive disability and fatigue were perceived as barriers to walking outdoors, which is similar to the findings for resuming valued activities [42]. Fatigue has also been identified in one study [44] that furthermore reported “shortness of breath” to be a barrier to physical activity. This is consistent with the findings in the present study where the respondents explicitly named fatigue, reduced aerobic capacity and the inability to walk long distances as barriers for outdoor walking. Interestingly, this perception of the relations between impaired body function, walking ability and outdoor walking seems consistent with quantitative research on the associations between community ambulation or physical activity in general and walking speed, physical fitness or balance [45, 46].

Finally, the opportunities that arise from participation are indicated as factors that determine outdoor walking. These findings are in line with the outcome of a recent review where intention and actual control over the behavior, the latter comparable with walking ability in the present study, were indicated as important in predicting physical activity [30].

The second aim of this study was to determine the usefulness of the PAD model to generate a comprehensive overview of barriers and facilitators. As a result of the integration of the ASE-model at the level of personal factors in the ICF, the PAD-model enables a comprehensive overview of barriers and facilitators for walking outdoors after stroke, as the ICF itself has not specifically coded personal factors [18, 30, 47]. However, to enable a deeper understanding of the meaning of social influence, self-efficacy and attitude from the ASE model it was necessary to explicit the underlying beliefs, i.e. normative, control and behavioral beliefs originating from the TPB, that underlies the ASE model. This is in line with the finding of Johnston and Dixon [30] that although the PAD model integrates psychological variables, i.e. the ASE model, it does not do so with a full behavioral model such as the TPB. Explicating the beliefs allowed us to achieve the comprehensive overview of barriers and facilitators for walking outdoors after stroke, that we aimed for.

Summarized, we were able to provide a comprehensive overview, addressing behavioral determinants along with physical and social determinants, that was lacking in the many earlier studies [48, 49]. We did not find significant differences from the facilitators and barriers that are already known to community ambulation aimed at improving participation or for exercise. Nor did we find significant differences between Dutch and the Anglo-Saxon populations in earlier studies. However, this underlines the validity of the barriers and facilitators that were identified by the respondents.

### Strengths and limitations

The use of the PAD-model as a directing concept allowed for a multidimensional description of barriers and facilitators for walking outdoors after stroke, giving insight into personal factors, environmental factors and behavioral mechanisms as well as constraints caused by body functions, limitations of activities and participation. The inclusion of respondents suffering from expressive aphasia and the use of focus-group interviews in addition to the individual interviews ensured saturation of the data and offered an opportunity validate the earlier collected data, increasing the validity of the outcomes and rigor of the study. Finally, all respondents were living in the community and the interviews were conducted at their homes, increasing the ecological validity of the study.

There were some limitations to the present study. First, most of the respondents were recruited from an existing network of physical therapists. They were either participating in exercise interventions or daycare interventions, including physical therapy, or did so in their rehabilitation past, which may have influenced their views on facilitators and barriers to walking outdoors. However, as eight respondents did not receive physical therapy at the time of the interviews it may be assumed that non-biased perceptions were also reported. Second, purposive sampling or inclusion criteria were not applied to the cognitive state of potential respondents. The reported prevalence of cognitive impairment in stroke varies from 20–80 % [50] indicating that in the sample of respondents in the present study cognitive impairment may have influenced the perceptions of facilitators and barriers to walking outdoors. However, as cognitive impairment is common after stroke it is plausible not to use it as an exclusion criterion. Third, convenience sampling regarding the focus-group interviews was challenging the diversity of the reported perceptions. Fortunately, the composition of the focus-groups proved similarly diverse to the group of respondents who participated in the individual interviews, allowing for the collection of rich data. Lastly, the researcher conducting the interviews had vast experience in working with individuals after stroke. This could challenge unbiased analysis of the data. However, as the analysis was triangulated with four other researchers this effect should have been only small.

## Conclusions

The PAD-model proved to be usable in displaying a comprehensive overview and insight in barriers and facilitators for outdoor walking in individuals after stroke and could support clinical reasoning and diagnostics in healthcare professionals. Specifically mapping environmental and personal factors as well as the domain of participation should receive adequate attention. It seems of particular importance to address social influence, e.g. care-givers’ or professionals’ influence, self-efficacy and attitude in the development of efficient interventions to influence the intention to walk outdoors. Furthermore, the improvement of walking ability and the creation of opportunities should be considered. As barriers and facilitators were reported in all domains of the PAD-model, the interventions that are provided by the healthcare professionals to stimulate outdoor walking should be tailored to fit specific needs, overcome barriers and make use of facilitators in each individual with stroke. This study shows that when developing research aimed at enhancing or further exploring underlying mechanisms for outdoor walking after stroke, the incorporation of behavioral, social, environmental as well as physical variables should be considered.

## Abbreviations

ASE, attitude, social influence and self-efficacy; FAC, functional ambulation categories; ICF, international classification of functioning, disability and health; PAD, physical activity in disability; TPB, theory of planned behavior; UCA, utrecht communication assessment; WHO, World Health Organization.
